# Supplementary material for: PRD-2 directly regulates casein kinase I and counteracts nonsense-mediated decay in the Neurospora circadian clock
Source: eLife. 2020 Dec 9;9:e64007. doi: 10.7554/eLife.64007 (PMC7746235; doi:10.7554/eLife.64007)
Supplement: Supplementary file 1. [file elife-64007-supp1.docx]

**Supplementary File 1. *Neurospora crassa* strains used in this study.**

| **Strain** | **Genotype** | **Source** |
| --- | --- | --- |
| FGSC2489 | OR74A *mat* A | FGSC |
| FGSC9718 | ∆*mus-51*::bar^R^; *mat* a | FGSC |
| FGSC11229 | ∆NCU04242::hph^R^; *mat* a | FGSC (*upf1^prd-6^*) |
| FGSC15706 | ∆NCU05267::hph^R^; ∆*mus-51*::bar^R^; *mat* a | FGSC (*upf2*) |
| FGSC11679 | ∆NCU03435::hph^R^; *mat* a | FGSC (*upf3*) |
| FGSC12475 | ∆NCU03775::hph^R^; *mat* A | FGSC |
| FGSC22441 | ∆NCU04187::hph^R^; *mat* a (*mus-51*^WT^) | FGSC (*cbp80*) |
| 87-3 | *ras-1^bd^*; *mat* a | This Laboratory |
| 328-4 | *ras-1^bd^*; *mat* A | This Laboratory |
| 613-43 | *ras-1^bd^*; *prd-2*; *mat* a | Feldman Laboratory |
| 613-102 | *ras-1^bd^*; *prd-2*; *mat* A | Feldman Laboratory |
| 1138-1 | [bar^R^::*qa-2*p-NCU00685]; ∆mus-52::hph^R^; *ras-1^bd^*; *mat* a [heterokaryon] | PMID: 19450520 |
| 1810 | *ras-1^bd^*; ∆NCU01019::bar^R^; *mat* A | This Study (Fig 1, 3, 4) |
| 834-1 | *ras-1^bd^*; hph^R^::*qa-2*p-NCU01019 | This Study (Fig 1) |
| 1929 | *csr-1*::NCU01019-luciferase; *ras-1^bd^*; *prd-2^INV^*; *mat* A | This Study (Fig 1) |
| 1930 | *csr-1*::NCU01019-luciferase; *ras-1^bd^*; *mat* A | This Study (Sup Fig 2) |
| 1786-1 | *csr-1*::*frq*_cbox_p-luciferase::bar^R^; *ras-1^bd^*; *mat* a | This Study (Fig 2, 4, 5) |
| 1931 | *csr-1*::*frq*_cbox_p-luciferase::bar^R^; *ras-1^bd^*; ∆NCU01019::bar^R^; *mat* A | This Study (Fig 2, 4, 5) |
| 1932 | *csr-1*::*frq*_cbox_p-luciferase::bar^R^; *ras-1^bd^*; V5-NCU01019∆SUZ[∆aa345-431]::hph^R^ | This Study (Fig 2) |
| 1933 | *csr-1*::*frq*_cbox_p-luciferase::bar^R^; *ras-1^bd^*; V5-NCU01019∆Cterminus[∆aa440-790]::hph^R^ | This Study (Fig 2) |
| 1934 | *csr-1*::*frq*_cbox_p-luciferase::bar^R^; ∆*mus-51*::bar^R^; NCU01019∆R3H[∆aa264-344]::hph^R^ | This Study (Fig 2) |
| CX002_B4-11-1 | *csr-1*::NCU01019∆SUZ[∆aa345-432]-V5::bar^R^; *ras-1^bd^*; ∆NCU01019::hph^R^ | This Study (Fig 2) |
| CX002_B4-11-6 | *csr-1*::NCU01019∆R3H[∆aa281-344]-V5::bar^R^; *ras-1^bd^*; ∆NCU01019::hph^R^ | This Study (Fig 2) |
| CX002_B4-11-5 | *csr-1*::NCU01019∆Cterminus[∆aa495-790]-V5::bar^R^; *ras-1^bd^*; ∆NCU01019::hph^R^ | This Study (Fig 2) |
| CX002_B4-11-3 | *csr-1*::NCU01019∆Cterminus[∆aa525-612]-V5::bar^R^; *ras-1^bd^*; ∆NCU01019::hph^R^ | This Study (Fig 2) |
| CX002_B4-11-8 | *csr-1*::NCU01019∆Cterminus[∆aa625-682]-V5::bar^R^; *ras-1^bd^*; ∆NCU01019::hph^R^ | This Study (Fig 2) |
| 1813 | *ras-1^bd^*; NCU01019-10xGly_V5_10xHis_3xFLAG::hph^R^; *mat* a | This Study (Fig 2, 3) |
| 1888 | *ras-1^bd^*; NCU16560-10xGly_V5_10xHis_3xFLAG::hph^R^ | This Study (Fig 3) |
| 1935 | bar^R^::*qa-2*p-NCU00685; *ras-1^bd^* | This Study (Fig 4, 5) |
| 1936 | bar^R^::*qa-2*p-NCU00685; *ras-1^bd^*; ∆NCU01019::bar^R^ | This Study (Fig 4) |
| 721-3 | NCU00685-SHORT-HA3::hph^R^; *ras-1^bd^*; *mat* a | This Study |
| 1937 | *csr-1*::*frq*_cbox_p-luciferase::bar^R^; NCU00685-SHORT-HA3::hph^R^; *ras-1^bd^*; *mat* a | This Study (Fig 4) |
| 1938 | *csr-1*::*frq*_cbox_p-luciferase::bar^R^; NCU00685-SHORT-HA3::hph^R^; *ras-1^bd^*; ∆NCU01019::bar^R^ | This Study (Fig 4) |
| 1939 | *csr-1*::*frq*_cbox_p-luciferase::bar^R^; *ras-1^bd^*; ∆NCU04242::hph^R^; *mat* A | This Study (Fig 5) |
| 1940 | *csr-1*::*frq*_cbox_p-luciferase::bar^R^; *ras-1^bd^*; ∆NCU01019::bar^R^; ∆NCU04242::hph^R^ | This Study (Fig 5) |
| 1941 | *csr-1*::*frq*_cbox_p-luciferase::bar^R^; *ras-1^bd^*; ∆NCU05267::hph^R^ | This Study (Fig 5) |
| 1942 | *csr-1*::*frq*_cbox_p-luciferase::bar^R^; *ras-1^bd^*; ∆NCU05267::hph^R^; ∆NCU01019::bar^R^ | This Study (Fig 5) |
| 1943 | *csr-1*::*frq*_cbox_p-luciferase::bar^R^; ∆NCU03435::hph^R^; *ras-1^bd^* | This Study (Fig 5) |
| 1944 | *csr-1*::*frq*_cbox_p-luciferase::bar^R^; ∆NCU03435::hph^R^; *ras-1^bd^*; ∆NCU01019::bar^R^ | This Study (Fig 5) |
| 1945 | bar^R^::*qa-2*p-NCU00685; *ras-1^bd^*; ∆NCU04242::hph^R^ | This Study (Fig 5) |
